# Supplementary material for: Hepatocyte-specific IL11 cis-signaling drives lipotoxicity and underlies the transition from NAFLD to NASH
Source: Nat Commun. 2021 Jan 4;12:66. doi: 10.1038/s41467-020-20303-z (PMC7782504; doi:10.1038/s41467-020-20303-z)
Supplement: Supplementary file 1 — Supplementary Information [file 41467_2020_20303_MOESM1_ESM.pdf]

SUPPLEMENTARY INFORMATION

Hepatocyte-specific IL11 cis-signaling drives lipotoxicity and underlies the transition from NAFLD to NASH

Authors: Jinrui Dong[1], Sivakumar Viswanathan[1], Eleonora Adami[1], Brijesh K. Singh[1], Sonia P. Chothani[1], Benjamin Ng[1,2], Wei Wen Lim[2], Jin Zhou[1], Madhulika Tripathi[1], Nicole SJ. Ko[1], Shamini G. Shekeran[1], Jessie Tan[1,2], Sze Yun Lim[2], Mao Wang[1], Pei Min Lio[2], Paul M. Yen[1], Sebastian Schafer[1,2], Stuart A. Cook[1,2,3,4*], Anissa A. Widjaja[1*].

*These authors jointly supervised this work.

*Corresponding author. Email: anissa.widjaja@duke-nus.edu.sg or stuart.cook@duke-nus.edu.sg

This file includes:
Supplementary Figures (1-11)
Supplementary Table 1

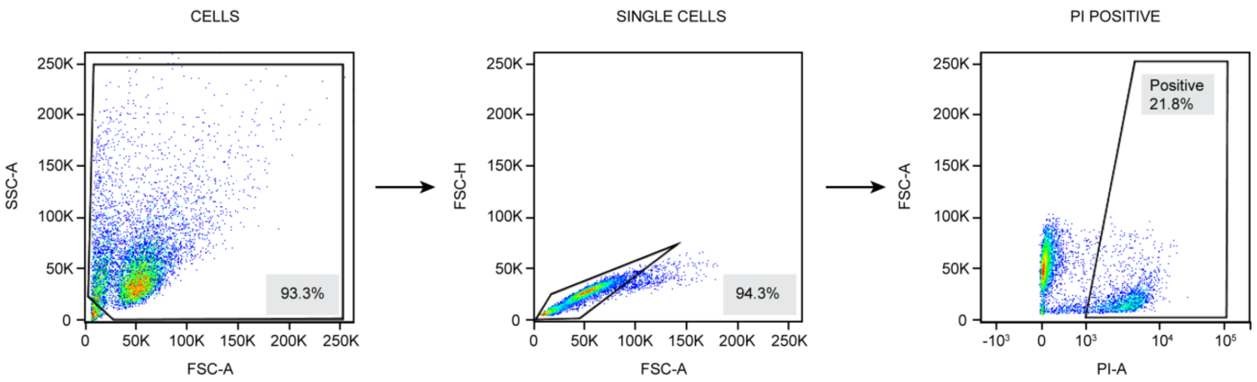

Supplementary Fig. 1 Gating Strategy for Propidium Iodide positive (PI^{+ve}) cells. The preliminary forward scatter (FSC)/side scatter (SSC) gates of the starting cell populations included 10,000 events. Debris (SSC-A vs FSC-A) and doublets (FSC-H vs FSC-A) were excluded. Boundaries between "positive" and "negative" staining were set at 10^3 for PI staining.

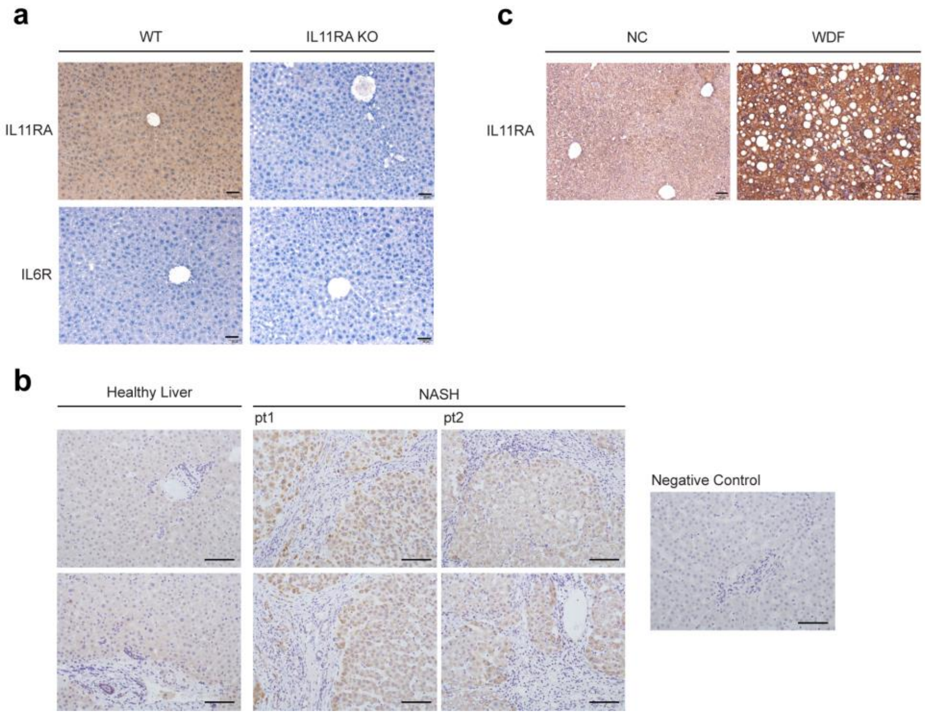

Supplementary Fig. 2 Expression of IL11RA in healthy and NASH livers. (**a**) Immunohistochemistry staining of IL11RA and IL6R in the livers of wildtype and *Il11ra*-deleted mice. Representative images from n=3 mice/group are shown. (**b**) Representative histological images from immunohistochemistry staining of IL11RA from control liver (n=3) and livers from patients (pt) (n=2) suffering from NASH with IL11RA antibody. Healthy control liver sections were stained without primary antibody as negative control (scale bars, 100µm). (**c**) Immunohistochemistry staining IL11RA in the livers of mice fed with normal chow diet (NC) or Western diet and Fructose (WDF) (scale bars, 50µm). Representative images from n=3 mice/group are shown.

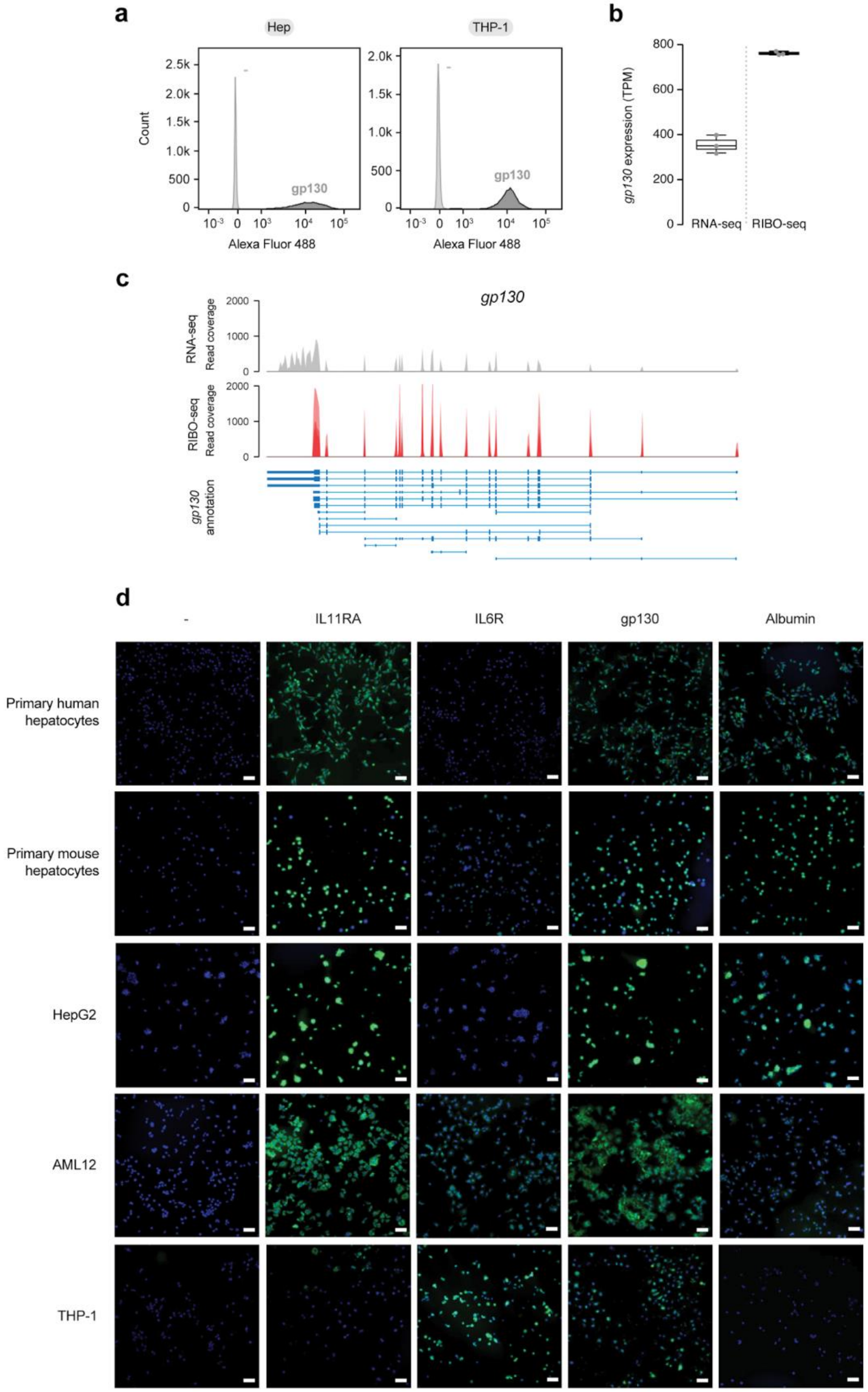
a
Hep
THP-1
Count
gp130
Alexa Fluor 488
b
gp130 expression (TPM)
RNA-seq
RIBO-seq
c
gp130
RNA-seq Read coverage
RIBO-seq Read coverage
gp130 annotation
d
-
IL11RA
IL6R
gp130
Albumin
Primary human hepatocytes
Primary mouse hepatocytes
HepG2
AML12
THP-1

Supplementary Fig. 3 Primary human hepatocytes highly express IL11RA but have limited IL6R. (**a**) Fluorescence intensity plots of negative control (-) and sgp130 staining on hepatocytes and THP-1. (**b**) *gp130* transcripts in primary human hepatocytes based on RNA-seq and Ribo-seq (TPM) (n=3). (**c**) Read coverage of *gp130* transcripts based on RNA-seq (gray) and Ribo-seq (red) of primary human hepatocytes (n=3). (**d**) Representative immunofluorescence images (scale bars, 100 µm) of IL11RA, IL6R, gp130, and Albumin expression in primary human hepatocytes, primary adult mouse hepatocytes, HepG2, AML12 and activated THP-1 cells (n=2/group) (**b**) Data are shown as box-and-whisker with median (middle line), 25th–75th percentiles (box) and min-max values (whiskers). Source data are provided as a Source Data file.

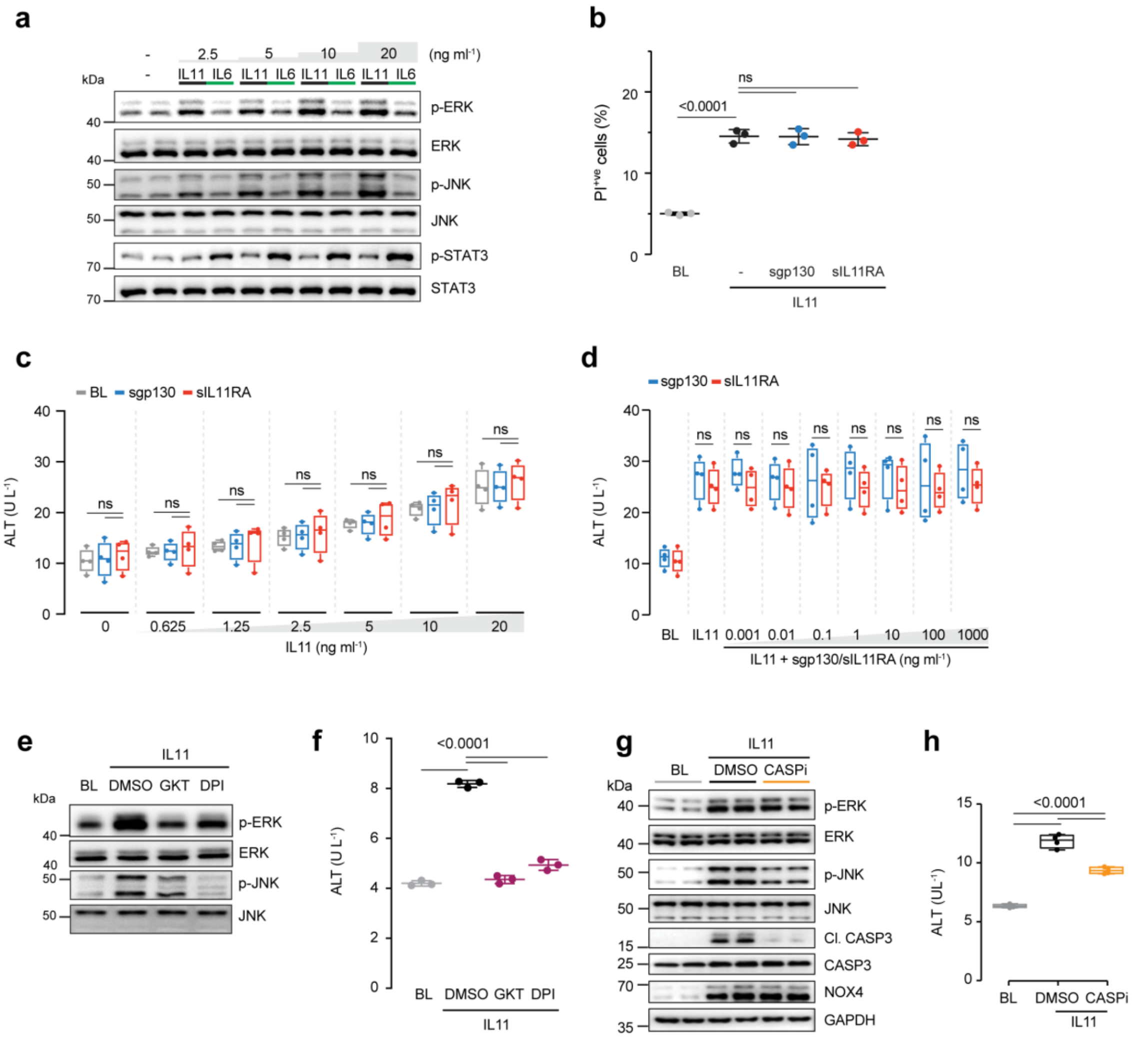

Supplementary Fig. 4 No evidence to support the existence of naturally occurring IL11 trans-signaling in hepatocytes. (**a**) Western blots showing ERK, JNK and STAT3 activation status by hepatocytes following a dose range stimulation of either IL11 or IL6. (**b**) Quantification of PI staining on IL11-stimulated hepatocytes (PI^{+ve} cells) in the presence of sgp130 or sIL11RA (n=3) shown in Fig. 2e. (**c**) Dose-dependent effect of increasing concentration of IL11 in the presence of sgp130 or sIL11RA on ALT levels secreted by hepatocytes (n=4). (**d**) Dose-dependent effect of increasing concentration of either sgp130 or sIL11RA on IL11-induced ALT secretion (n=4). Effect of NOX4 inhibitors (GKT-13781 and diphenyleneiodonium chloride (DPI)) on (**e**) ERK and JNK activation and on (**f**) ALT secretion (n=3) from IL11-stimulated hepatocytes. (**g**) NOX4 expression and activation status of ERK, JNK, and caspase-3 (n=2/group), and (**h**) ALT levels in the supernatant (n=4) from IL11-stimulated hepatocytes in the presence of either DMSO or pan-caspase inhibitor (Z-VAD-FMK). (**a-h**) Primary human hepatocytes; 24 hours stimulation. (**b-h**) IL11 (20 ng/ml), sgp130 and sIL11RA (1 µg/ml), DPI (10 µM), GKT-13781 (20 µM), Z-VAD-FMK (20 µM), unless otherwise stated in the figure. (**a**

and **e**) Representative data from n=2 independent experiments are shown. (**b**, **f**) Data are shown as mean ± SD; (**c**, **d**, **h**) data are shown as box-and-whisker with median (middle line), 25th–75th percentiles (box) and min-max values (whiskers); (**b**-**d**, **f**, **h**) One-way ANOVA with Tukey’s correction. Source data are provided as a Source Data file.

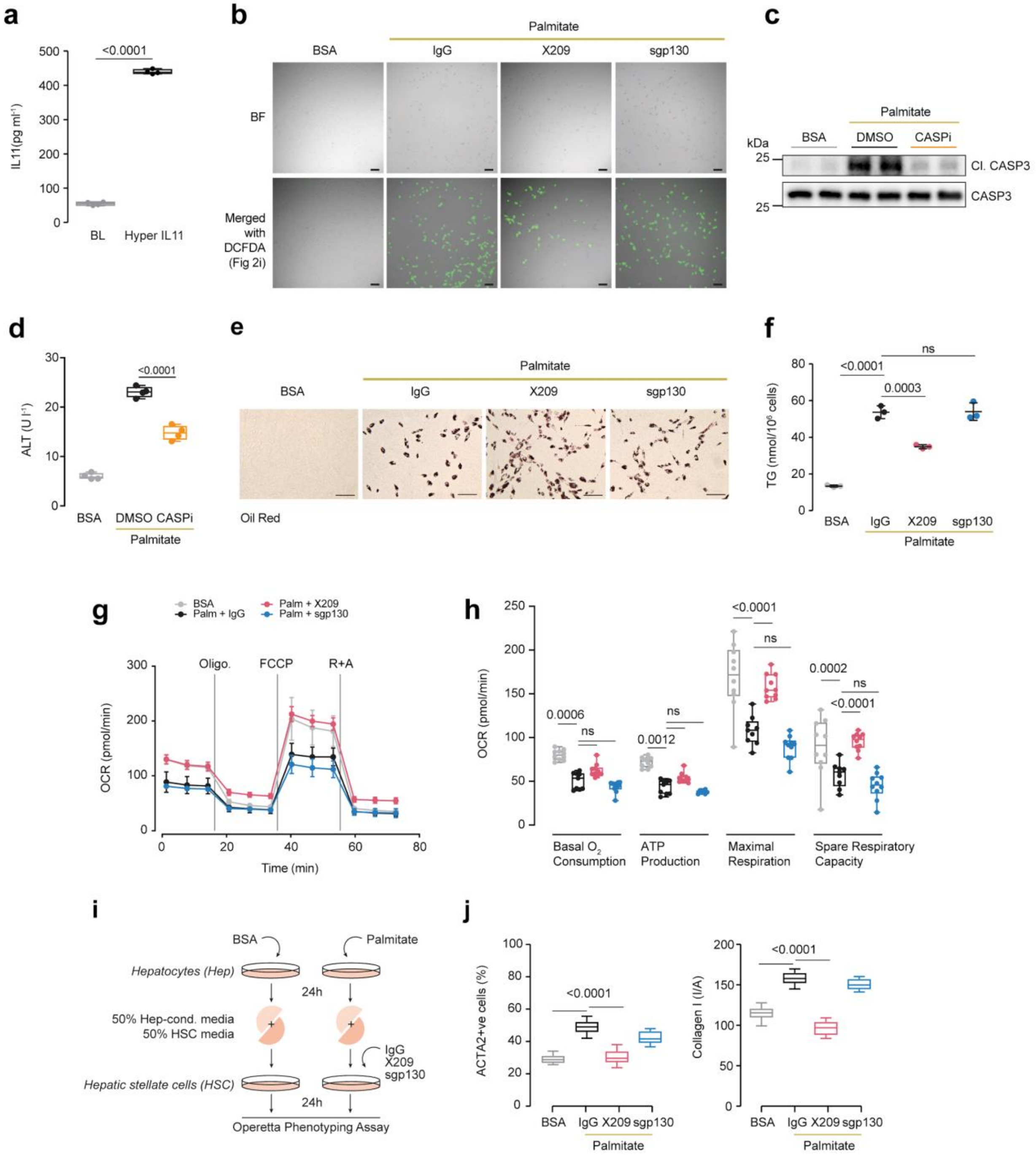

Supplementary Fig. 5 Metabolic effects of autocrine IL11 signaling in lipotoxic hepatocytes and pro-fibrotic paracrine effects on hepatic stellate cells. (**a**) ELISA of IL11 expression after hyperIL11 stimulation (n=4). (**b**) Representative brightfield images and merged fluorescence-brightfield images (scale bars, 100 µm) for DCFDA staining experiment shown in Fig. 2i. Effect of pan-caspase inhibitor on (**c**) caspase-3

cleavage and (**d**) ALT secretion (n=4) following palmitate treatment. (**e**) Representative images of Oil Red O staining (scale bars, 100 µm). (**f**) Triglyceride levels in IgG, X209, or sgp130-treated palmitate-stimulated hepatocytes (n=3). (**g**-**h**) Seahorse assay showing (**g**) mitochondrial oxygen consumption rate (OCR) and (**h**) the changes in OCR during basal oxygen consumption, ATP production, maximum respiratory capacity and spare respiratory capacity (BSA, Palm+X209, Palm+sgp130, n=10/group; Palm+IgG, n=9/group). (**i**) Schematic of media transfer experiments in which conditioned media from BSA or palmitate-stimulated hepatocyte (24 hours) were used to treat primary human hepatic stellate cells (HSCs) in the presence of either IgG, X209 or sgp130. (**j**) Quantification of $ACTA2^{+ve}$ cells and Collagen 1 immunostaining of HSCs treated with conditioned media from palmitate-stimulated hepatocytes as shown in Fig. 2m (n=14). (**a**-**j**) Primary human hepatocytes, (**i**-**j**) primary human hepatic stellate cells; 24 hours stimulation; hyperIL11 (20 ng/ml), palmitate (0.5 mM), IgG (2 µg/ml), anti-IL11 (X203, 2 µg/ml), anti-IL11RA (X209, 2 µg/ml), or sgp130 (1 µg/ml), pan-Caspase inhibitor (Z-VAD-FMK, 20 µM). (**a**, **f**) Mean$\pm$SD; (**d**, **h**, **j**) data are shown as box-and-whisker with median (middle line), 25th–75th percentiles (box) and min-max values (whiskers). (**a**) 2-tailed Student’s *t*-test; (**d**, **f**, **h**, **j**) One-way ANOVA with Tukey’s correction. (**b** and **e**) Representative dataset from n=4 independent experiment is shown. Source data are provided as a Source Data file.

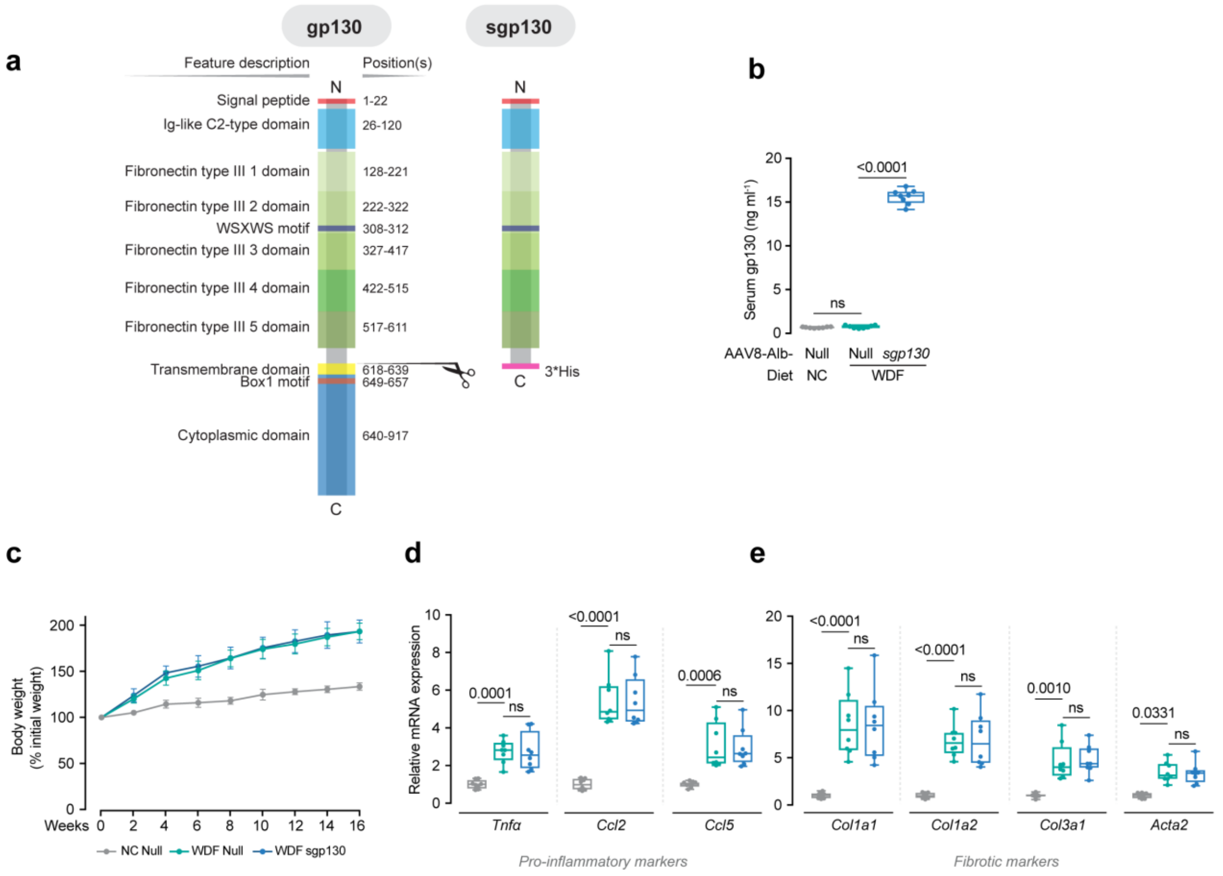

Supplementary Fig. 6 Soluble gp130 expression does not protect mice from WDF-induced obesity or expression of fibro-inflammatory genes in the liver. (**a**) Schematic of gp130 protein domain structure and its amino acid position (left) and the domains that were used to construct sgp130 in this study (right). (**b**-**e**) Data for WDF-sgp130 *in vivo* experiments as shown in **Fig. 3a** (n=8 mice/group). (**b**) Serum gp130 levels in NC-fed control mice and WDF-fed AAV8-Alb-Null- and AAV8-Alb-sgp130-injected mice. (**c**) Effect of 16 weeks of WDF on body weight of AAV8-Alb-Null- and AAV8-Alb-sgp130-injected mice. Data are shown as mean ± SD. (**d**-**e**) Hepatic mRNA expression of (**d**) pro-inflammatory markers (*Tnf*α, *Ccl2*, *Ccl5*) and (**e**) fibrosis markers (*Col1a1, Col1a2, Col3a1, Acta2*) as shown in Fig. 3o. (**b**, **d**-**e**) Data are shown as box-and-whisker with median (middle line), 25th–75th percentiles (box) and min-max values (whiskers); one-way ANOVA with Tukey's correction. Source data are provided as a Source Data file.

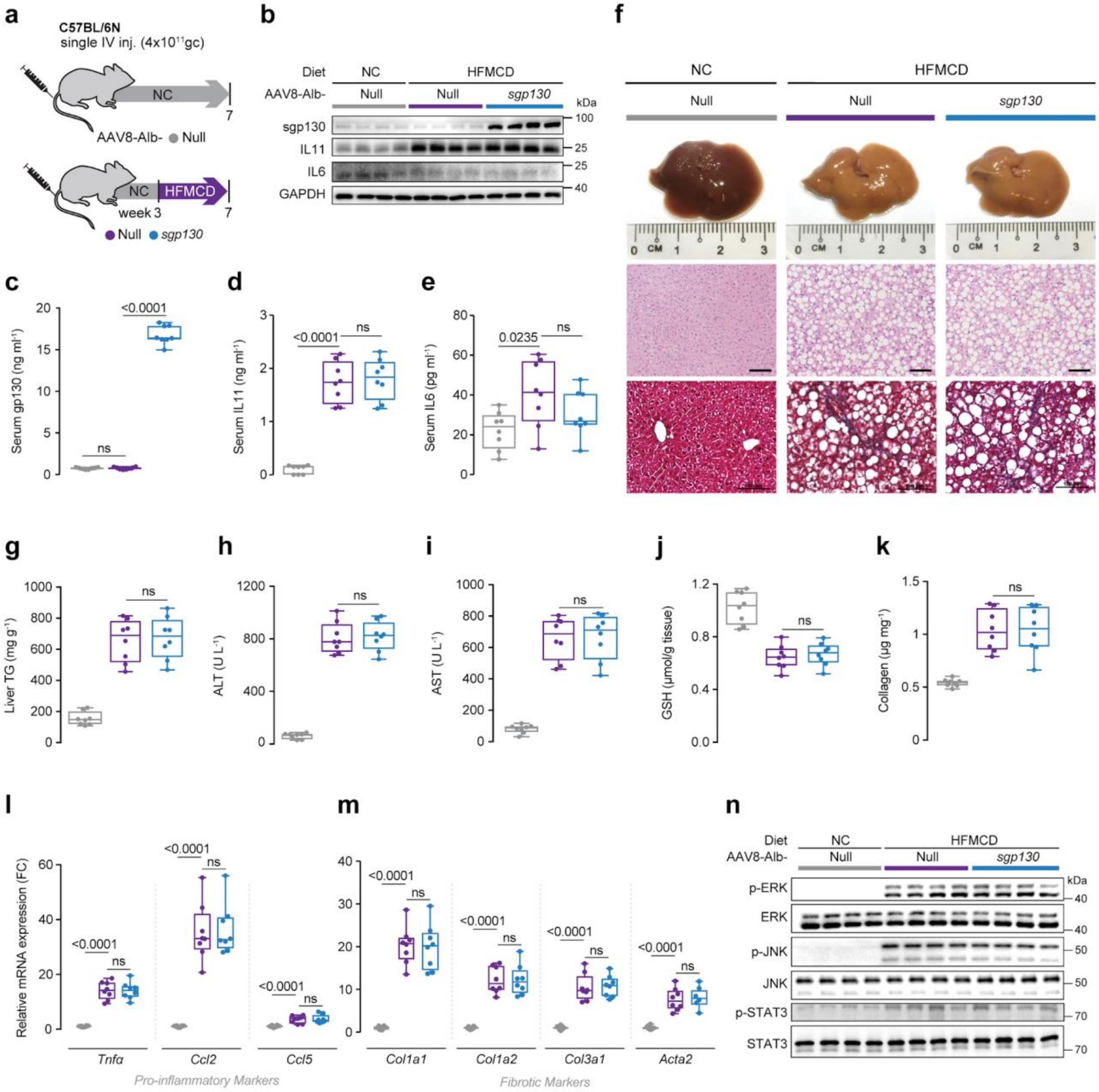

Supplementary Fig. 7 Inhibition of putative trans-signaling of IL6 family members has no effect on NASH phenotypes in mice on HFMCD diet. (**a**) Schematic of mice with hepatocyte-specific expression of sgp130 in mice on HFMCD diets for data shown in B-N. Mice were intravenously injected with either AAV8-Alb-Null or AAV8-Alb-sgp130 and fed HFMCD for 4 weeks. (**b**) Western blots showing hepatic levels of sgp130, IL11 and IL6 with GAPDH shown as internal control (n=4 mice/group). (**c**) Serum gp130 levels. (**d**) Serum IL11 levels. (**e**) Serum IL6 levels. (**f**) Representative gross anatomy, H&E-stained (scale bars, 50 µm) and Masson's Trichrome (scale bars, 100 µm) images of livers. Representative dataset from n=8 mice/group is shown for gross anatomy; representative dataset from n=4 mice/group is shown for H&E-stained and Masson's Trichrome images. (**g**) Hepatic triglycerides content. (**h**) Serum ALT levels. (**i**) Serum

AST levels. (**j**) Hepatic GSH content. (**k**) Hepatic collagen levels. (**l**, **m**) Hepatic mRNA expression of (**l**) pro-inflammatory markers (*Tnf*α, *Ccl2*, *Ccl5*) and (**m**) fibrosis markers (*Col1a1, Col1a2, Col3a1, Acta2*). (**n**) Western blots of hepatic p-ERK, ERK, p-JNK, JNK, p-STAT3, STAT3 (n=4 mice/group). (**c**-**e**, **g**-**m**) Data are shown as box-and-whisker with median (middle line), 25th–75th percentiles (box) and min-max values (whiskers); one-way ANOVA with Tukey's correction; n=8 mice/group. Source data are provided as a Source Data file.

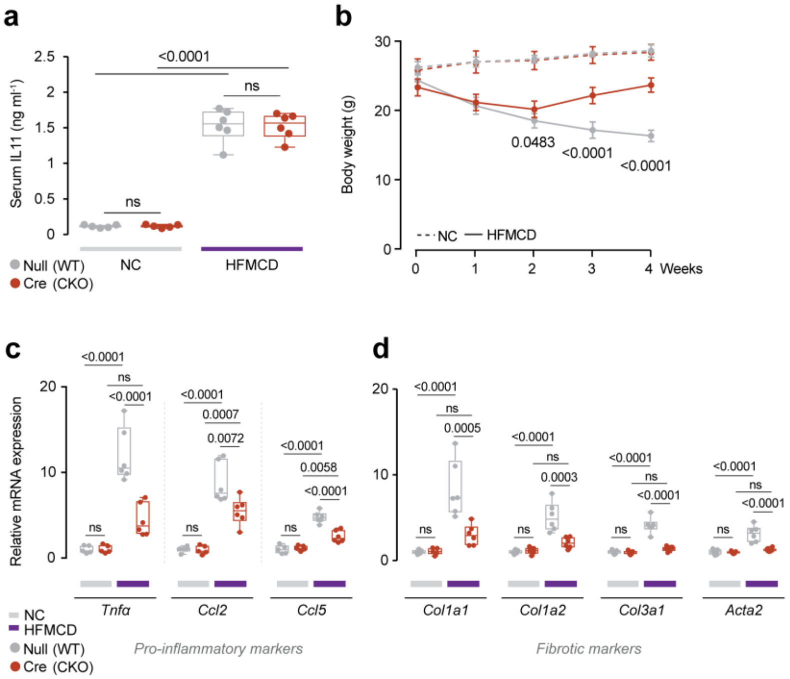

Supplementary Fig. 8 Mice with hepatocyte-specific deletion of *Il11ra1* are protected from HFMCD-induced weight loss and upregulation of fibro-inflammatory genes in the liver. (**a**-**d**) Data for control and CKO mice on NC and HFMCD diet as shown in **Fig. 4a**. (**c**-**d**) Hepatic mRNA expression of (**c**) pro-inflammatory markers (*Tnf*α, *Ccl2*, *Ccl5*) and (**d**) fibrotic markers (*Col1a1, Col1a2, Col3a1, Acta2*) from control and CKO mice on NC and HFMCD diet as shown in Fig. 4j. (**a**, **c**, **d**) Data are shown as box-and-whisker with median (middle line), 25th–75th percentiles (box) and min-max values (whiskers); (**b**) data are shown as mean$\pm$SD; 2-way ANOVA with Tukey's correction. (**a**-**d**) NC, n=5 mice/group; HFMCD, n=6 mice/group. Source data are provided as a Source Data file.

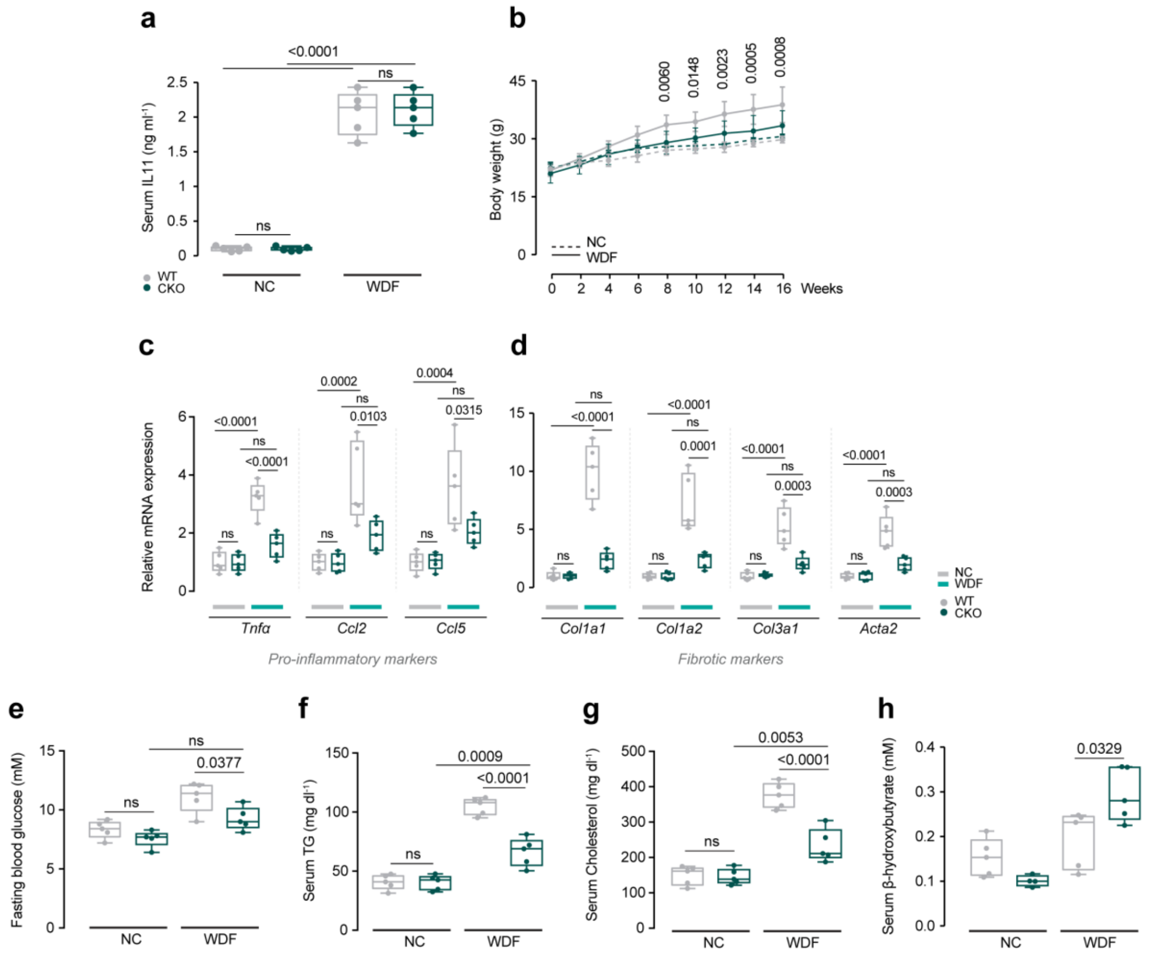

Supplementary Fig. 9 Hepatocyte-specific *Il11ra1* deleted mice are protected from WDF-induced obesity and hepatic and systemic NASH phenotypes. (**a**-**i**) Data for control and CKO mice on NC and WDF diet as shown in Fig. 5a. (**a**) Serum IL11 levels. (**b**) Body weight of mice. (**c**-**d**) Hepatic mRNA expression of (**c**) pro-inflammatory markers (*Tnf*α, *Ccl2*, *Ccl5*) and (**d**) fibrotic markers (*Col1a1, Col1a2, Col3a1, Acta2*) as shown in Fig. 5l. (**e**) Fasting blood glucose levels. (**f**) Serum triglycerides levels. (**g**) Serum cholesterol levels. (**h**) Serum β-hydroxybutyrate levels. (**a**, **c**-**h**) Data are shown as box-and-whisker with median (middle line), 25th–75th percentiles (box) and min-max values (whiskers); (**b**) data are shown as mean$\pm$SD. (**a**-**h**) 2-way ANOVA with Tukey's correction. N=5 mice/group except for (H) NC CKO (n=4 mice/group). Source data are provided as a Source Data file.

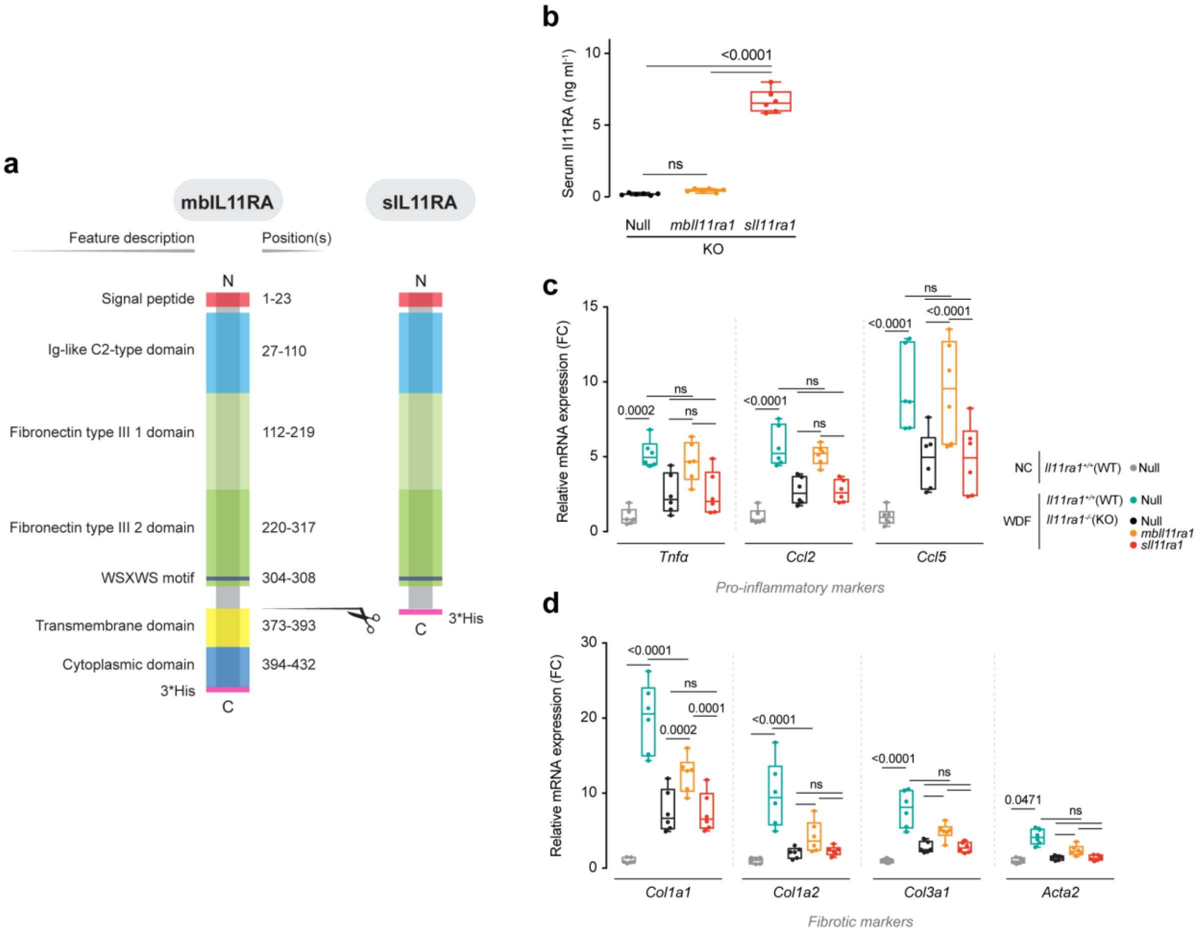

Supplementary Fig. 10 Hepatocyte-specific IL11 cis-signaling but not IL11 trans-signaling drives WDF-induced steatohepatitis in mice. (**a**) Schematic of full-length membrane-bound IL11RA protein domain structure and its amino acid position (left) and the domains that were used to construct soluble IL11RA (right). (**b**-**d**) Data for WDF feeding regimen on *Il11ra1*$^{+/+}$ (WT) mice and mice globally deleted for *Il11ra* (*Il11ra1*$^{-/-}$; KO mice) that had been injected with AAV8-Alb-Null, AAV8-Alb-mbIl11ra1 (full length membrane-bound Il11ra1) or AAV8-Alb-sIl11ra1 (soluble form of Il11ra1) as illustrated in Fig. 6a. (**b**) Serum IL11RA levels in AAV8-Alb-Null, AAV8-Alb-mbIl11ra, and AAV8-Alb-sIl11ra1-injected KO mice on WDF. (**c**-**d**) Hepatic mRNA expression of (**c**) pro-inflammatory markers (*Tnfα*, *Ccl2*, *Ccl5*) and (**d**) fibrotic markers (*Col1a1, Col1a2, Col3a1, Acta2*). (**b**-**d**) Data are shown as box-and-whisker with median (middle line), 25th–75th percentiles (box) and min-max values (whiskers); one-way ANOVA with Tukey's correction; n=6 mice/group. Source data are provided as a Source Data file.

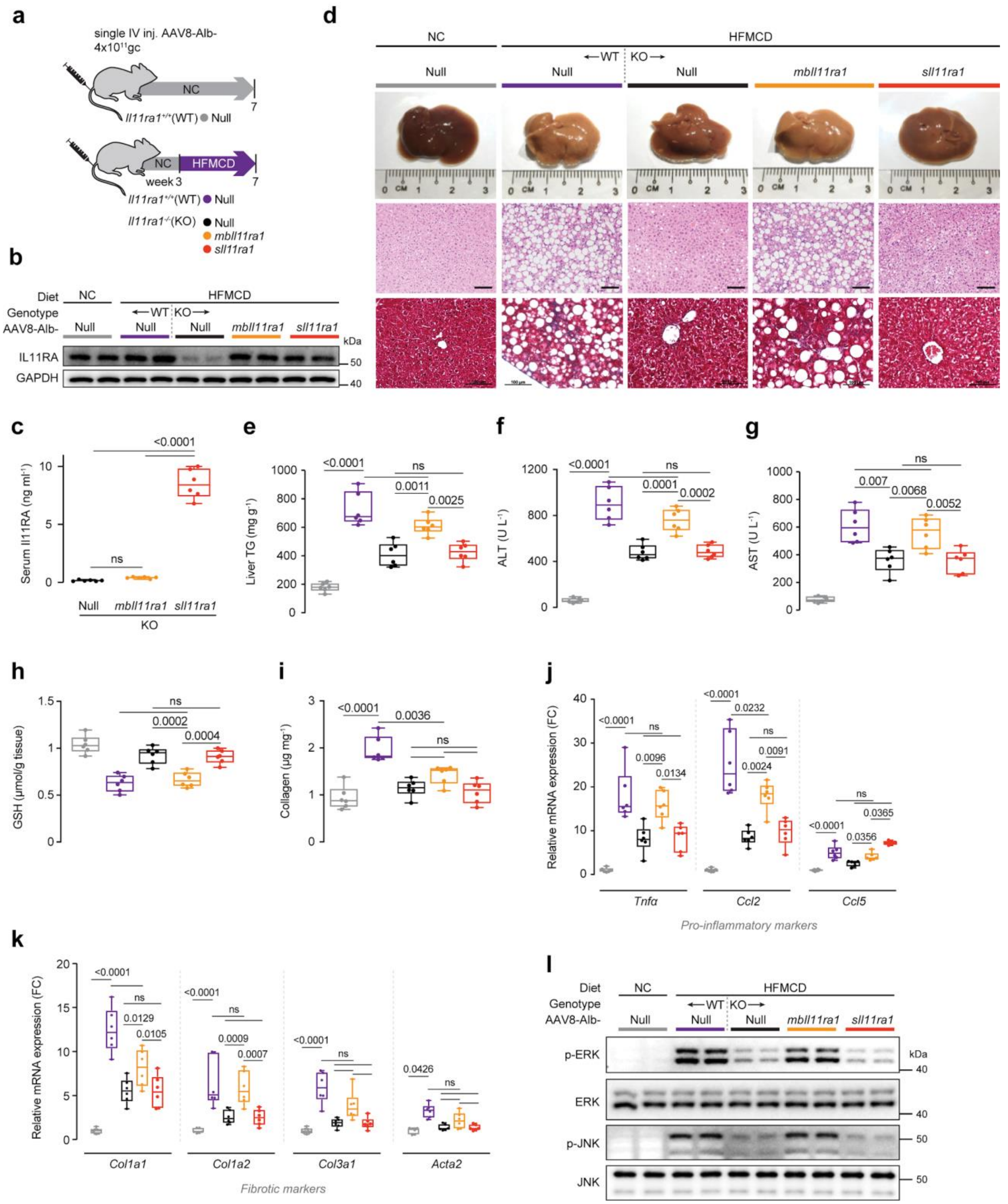

Supplementary Fig. 11 Hepatocyte-specific IL11 cis-signaling but not IL11 trans-signaling drives steatohepatitis in mice on a HFMCD. (**a**) Schematic of HFMCD-fed WT and KO mice for experiments shown in **b**-**l**. KO mice were intravenously injected

with either AAV8-Alb-Null, AAV8-Alb-mbIl11ra1 or AAV8-ALB-sIl11ra1; WT mice received AAV8-Alb-Null as control. Three weeks following virus administration, mice were started on HFMCD feeding for 4 weeks. (**b**) Western blots showing hepatic levels of IL11RA and GAPDH. (**c**) Serum IL11RA levels. (**d**) Representative gross anatomy, H&E-stained (scale bars, 50 μm) and Masson's Trichrome (scale bars, 100 μm) images of livers. Representative dataset from n= mice/group is shown for gross anatomy; representative dataset from n=4 mice/group is shown for H&E-stained and Masson's Trichrome images. (**e**) Hepatic triglycerides content. (**f**) Serum ALT levels. (**g**) Serum AST levels. (**h**) Hepatic GSH levels. (**i**) Hepatic collagen content. (**j**-**k**) Hepatic mRNA expression of (**j**) pro-inflammatory markers (*Tnf*α, *Ccl2*, *Ccl5*) and (**k**) fibrotic markers (*Col1a1, Col1a2, Col3a1, Acta2*). (**l**) Western blots showing activation status of hepatic ERK and JNK. (**c**, **e**-**k**) Data are shown as box-and-whisker with median (middle line), 25th–75th percentiles (box) and min-max values (whiskers); one-way ANOVA with Tukey's correction; n=6 mice/group. Source data are provided as a Source Data file.

Supplementary Table 1. Primer sequences for RT-qPCR

Host	Gene	Forward (5'-3')	Reverse (5'-3')
Mouse	*Tnf*α	ATGAGAAGTTCCCAAATGGC	CTCCACTTGGTGGTTTGCTA
	Ccl2	GAAGGAATGGGTCCAGACAT	ACGGGTCAACTTCACATTCA
	Ccl5	GCTGCTTTGCCTACCTCTCC	TCGAGTGACAAACACGACTGC
	Col1a1	GAGCAGACGGGAGTTTCTCCT	CATGTAGACTCTTTGCGGCTG
	Col1a2	AGGATTGGTCAGAGCAGTGT	TCCACAACAGGTGTCAGGGT
	Col3a1	CCACCCAATACAGGTCAAATGC	TGAGTATGACCGTTGCTCTGC
	Acta2	TCCATCGTCCACCGCAAAT	GCCAGGGCTACAAGTTAAGG
	Gapdh	CTGGAAAGCTGTGGCGTGAT	GACGGACACATTGGGGGTAG
