# Supplementary material for: Hepatocyte-specific IL11 cis-signaling drives lipotoxicity and underlies the transition from NAFLD to NASH
Source: Nat Commun. 2021 Jan 4;12:66. doi: 10.1038/s41467-020-20303-z (PMC7782504; doi:10.1038/s41467-020-20303-z)
Supplement: Supplementary file 3 — Reporting Summary [file 41467_2020_20303_MOESM3_ESM.pdf]

nature research

Corresponding author(s): Anissa A Widjaja and Stuart A Cook

Last updated by author(s): Oct 27, 2020

Reporting Summary

Nature Research wishes to improve the reproducibility of the work that we publish. This form provides structure for consistency and transparency in reporting. For further information on Nature Research policies, see our Editorial Policies and the Editorial Policy Checklist.

Statistics

For all statistical analyses, confirm that the following items are present in the figure legend, table legend, main text, or Methods section.

n/a	Confirmed	
☐	☒	The exact sample size (*n*) for each experimental group/condition, given as a discrete number and unit of measurement
☐	☒	A statement on whether measurements were taken from distinct samples or whether the same sample was measured repeatedly
☐	☒	The statistical test(s) used AND whether they are one- or two-sided<br>*Only common tests should be described solely by name; describe more complex techniques in the Methods section.*
☒	☐	A description of all covariates tested
☐	☒	A description of any assumptions or corrections, such as tests of normality and adjustment for multiple comparisons
☐	☒	A full description of the statistical parameters including central tendency (e.g. means) or other basic estimates (e.g. regression coefficient) AND variation (e.g. standard deviation) or associated estimates of uncertainty (e.g. confidence intervals)
☐	☒	For null hypothesis testing, the test statistic (e.g. *F*, *t*, *r*) with confidence intervals, effect sizes, degrees of freedom and *P* value noted<br>*Give P values as exact values whenever suitable.*
☒	☐	For Bayesian analysis, information on the choice of priors and Markov chain Monte Carlo settings
☒	☐	For hierarchical and complex designs, identification of the appropriate level for tests and full reporting of outcomes
☒	☐	Estimates of effect sizes (e.g. Cohen's *d*, Pearson's *r*), indicating how they were calculated

Our web collection on statistics for biologists contains articles on many of the points above.

Software and code

Policy information about availability of computer code

Data collection	The study did not involve software and code for data collection.
Data analysis	We used standard software throughout the manuscript (GraphPad Prism version 6.07) that is publicly available. FlowJo version X software (TreeStar) was used for data analysis of flow cytometry.

For manuscripts utilizing custom algorithms or software that are central to the research but not yet described in published literature, software must be made available to editors and reviewers. We strongly encourage code deposition in a community repository (e.g. GitHub). See the Nature Research guidelines for submitting code & software for further information.

Data

Policy information about availability of data

All manuscripts must include a data availability statement. This statement should provide the following information, where applicable:

- Accession codes, unique identifiers, or web links for publicly available datasets
- A list of figures that have associated raw data
- A description of any restrictions on data availability

All data are available within the Article or Supplementary Information. The RNA-seq and RIBO-seq data reported in this paper are available in NCBI BioProject ID: PRJNA670552. Source data are provided with this paper.

Field-specific reporting

Please select the one below that is the best fit for your research. If you are not sure, read the appropriate sections before making your selection.

- [x] Life sciences
- [ ] Behavioural & social sciences
- [ ] Ecological, evolutionary & environmental sciences

For a reference copy of the document with all sections, see nature.com/documents/nr-reporting-summary-flat.pdf

Life sciences study design

All studies must disclose on these points even when the disclosure is negative.

Sample size	Sample sizes were based on the experience of the authors with molecular and invivo studies as published in many studies.<br>For animal models, experiments were designed to detect differences between treatment groups or genotype-dependent effects at 80% power (α=0.05).<br>Sample sizes may vary depending on animal availability<br>Sample size for cell based assays were determined based on sample availability.
Data exclusions	No data was excluded from the analyses.
Replication	All experiments were repeated with reproducibility. The replication number for each experiment is indicated in the legend of the corresponding figure.
Randomization	For in vitro studies, the cells from each cell line required for all tested conditions were pooled, equal number of cell were then seeded and stimulated/treated randomly. For in vivo studies, the animal were allocated to experimental groups to ensure equal litter/sex/age across groups.
Blinding	For in vitro experiments, investigators were not blinded to group allocation during data collection and analysis. For in vivo experiments, treatments/genotypes were not disclosed to investigators generating quantitative readouts during data collection but investigators were not blinded during data analysis.

Reporting for specific materials, systems and methods

We require information from authors about some types of materials, experimental systems and methods used in many studies. Here, indicate whether each material, system or method listed is relevant to your study. If you are not sure if a list item applies to your research, read the appropriate section before selecting a response.

Materials & experimental systems

n/a	Involved in the study	
	x	Antibodies
	x	Eukaryotic cell lines
x		Palaeontology and archaeology
	x	Animals and other organisms
x		Human research participants
x		Clinical data
x		Dual use research of concern

Methods

n/a	Involved in the study	
x		ChIP-seq
	x	Flow cytometry
x		MRI-based neuroimaging

Antibodies

Antibodies used	ACTA2 (ab7817, Abcam, 1:1000 for western blot and 1:500 operetta assay), Albumin (ab207327, Abcam, 1:100 for IF and flow cytometry), Cleaved Caspase-3 (9664, CST, 1:1000), Caspase-3 (9662, CST, 1:1000), Collagen I (ab34710, Abcam, 1:500), phospho-ERK1/2 (4370, CST, 1:1000), ERK1/2 (4695, CST, 1:1000), GAPDH (2118, CST, 1:1000), gp130 (human, PA5-28932, Thermo Fisher, 1:100), gp130 (mouse, PA5-99526, Thermo Fisher, 1:100), gp130 (extracellular, PA5-77476, Thermo Fisher, 1:1000), IgG (11E10, Aldevron), IL6 (AF506, R&D systems, 1:1000), IL6R (flow cytometry, ab222101, Abcam, 1:100), IL6R (human, IHC and IF, MA1-80456, Thermo Fisher, 1:100), IL6R (mouse, IF, ab83053, Abcam, 1:100), IL11 (X203, Aldevron), IL11RA (inhibition study, X209, Aldevron), IL11RA (IHC, IF, flow cytometry, ab125015, Abcam), IL11RA (western blot, sc-130920, Santa Cruz, 1:200), phospho-JNK (4668, CST, 1:1000), JNK (9252, CST, 1:1000), NOX4 (MA5-32090, Invitrogen, 1:1000), phospho-STAT3 (4113, CST, 1:1000), STAT3 (4904, CST, 1:1000), mouse Alexa Fluor 488 secondary antibody (ab150113, Abcam, 1:200), mouse HRP (7076, CST, 1:2000), rabbit Alexa Fluor 488 secondary antibody (ab150077, Abcam, 1:200), rabbit HRP (7074, CST, 1:2000), rat Alexa Fluor 488 secondary antibody (ab150157, Abcam, 1:200), rat HRP (31470, Santa Cruz, 1:800).
Validation	All antibodies used are commercially available and validated by the manufacturers, as indicated on the respective websites of each commercial vendor. Please refer to the commercial website of each primary antibody for more details:<br>(1) ACTA2: human, mouse; western blot, IF. (https://www.abcam.com/alpha-smooth-muscle-actin-antibody-1a4-ab7817.html)<br>(2) Albumin: human, mouse; IF, flow cytometry. (https://www.abcam.com/albumin-antibody-epr20195-ab207327.html)

(3) Cleaved Caspase-3: human, mouse; western blot. (https://www.cellsignal.com/products/primary-antibodies/cleaved-caspase-3-asp175-5a1e-rabbit-mab/9664?Ntk=Products&Ntt=9664)
(4) Caspase-3: human, mouse; western blot. (https://www.cellsignal.com/products/primary-antibodies/caspase-3-antibody/9662?Ntk=Products&Ntt=9662)
(5) Collagen I: human; IF. (https://www.abcam.com/collagen-i-antibody-ab34710.html)
(6) phospho-ERK1/2: human, mouse; western blot. (https://www.cellsignal.com/products/primary-antibodies/phospho-p44-42-mapk-erk1-2-thr202-tyr204-d13-14-4e-xp-rabbit-mab/4370?Ntk=Products&Ntt=4370)
(7) ERK1/2: human, mouse; western blot. (https://www.cellsignal.com/products/primary-antibodies/p44-42-mapk-erk1-2-137f5-rabbit-mab/4695?Ntk=Products&Ntt=4695)
(8) GAPDH: human, mouse; western blot. (https://www.cellsignal.com/products/primary-antibodies/gapdh-14c10-rabbit-mab/2118?Ntk=Products&Ntt=2118)
(9) gp130: human, mouse; flow cytometry, IF. (https://www.thermofisher.com/antibody/product/GP130-Antibody-Polyclonal/PA5-99526)
(10) gp130 (extracellular): mouse; western blot. (https://www.thermofisher.com/antibody/product/GP130-extracellular-Antibody-Polyclonal/PA5-77476)
(11) IL6: mouse; western blot. (https://www.rndsystems.com/products/rat-il-6-antibody_af506)
(12) IL6R: human; flow cytometry. (https://www.abcam.com/il-6r-antibody-epr22628-236-ab222101.html)
(13) IL6R: human; IHC, IF. (https://www.thermofisher.com/antibody/product/IL-6-Receptor-Antibody-clone-B-R6-Monoclonal/MA1-80456)
(14) IL6R: mouse; flow cytometry, IHC, IF. (https://www.abcam.com/il-6r-antibody-ap-mab0848-ab83053.html)
(15) IL11RA: human; flow cytometry, IHC, IF. (https://www.abcam.com/il-11ra-antibody-epr5446-ab125015.html)
(16) IL11RA: human, mouse; western blot, IHC, IF. (https://www.scbt.com/p/il-11ralpha-antibody-4d12)
(17) phospho-JNK: mouse; western blot. (https://www.cellsignal.com/products/primary-antibodies/phospho-sapk-jnk-thr183-tyr185-81e11-rabbit-mab/4668?Ntk=Products&Ntt=4668)
(18) JNK: mouse; western blot. (https://www.cellsignal.com/products/primary-antibodies/sapk-jnk-antibody/9252?Ntk=Products&Ntt=9252)
(19) NOX4: mouse; western blot. (https://www.thermofisher.com/antibody/product/NOX4-Antibody-clone-SY0214-Recombinant-Monoclonal/MA5-32090)
(20) phospho-STAT3: mouse; western blot. (https://www.cellsignal.com/products/primary-antibodies/phospho-stat3-tyr705-m9c6-mouse-mab/4113?Ntk=Products&Ntt=4113)
(21) STAT3: mouse; western blot. (https://www.cellsignal.com/products/primary-antibodies/stat3-79d7-rabbit-mab/4904?Ntk=Products&Ntt=4904)

Eukaryotic cell lines

Policy information about cell lines

Cell line source(s)

Primary human hepatocytes (5200, ScienCell)
Primary mouse hepatocytes (ABC-TC3928, AcceGen Biotech)
Primary human hepatic stellate cells (5300, ScienCell)
HepG2 (ATCC)
AML12 (ATCC)
THP-1 (ATCC)

Authentication

Primary human hepatocytes and primary human hepatic stellate cells were previously authenticated by ScienCell. Primary mouse hepatocytes were previously authenticated by AcceGen Biotech. HepG2, AML12, and THP-1 cells were previously authenticated by ATCC.

Mycoplasma contamination

All cell lines were tested to be free of mycoplasma contamination.

Commonly misidentified lines
(See ICLAC register)

No commonly misidentified cell lines were used in the study.

Animals and other organisms

Policy information about studies involving animals; ARRIVE guidelines recommended for reporting animal research

Laboratory animals

Mice were housed in temperatures of 21-24°C with 40-70% humidity on a 12 h light/12 h dark cycle and provided with food and water ad libitum.
Mouse models of metabolic liver disease
HFMCD
6-8 weeks old C57BL/6N, Il11ra1-/- mice, and Il11ra1loxP/loxP and their respective control were fed with methionine- and choline-deficient diet supplemented with 60 kcal% fat (HFMCD, A06071301B16, Research Diets) for 4 weeks. Control mice received normal chow (NC, Specialty Feeds).
WDF
6-8 weeks old C57BL/6N, Il11ra1-/- mice, and Il11ra1loxP/loxP and their respective control were fed western diet (D12079B, Research Diets) supplemented with 15% weight/volume fructose in drinking water (WDF) for 16 weeks. Control mice received NC and tap water.
Il11ra1-deleted mice (KO)
6-8-week old male Il11ra1-/- mice (B6.129S1-Il11ratm1Wehi/J, Jackson's Laboratory) were intravenously injected with 4X1011 genome copies (gc) of AAV8-Alb-mbIl11ra1 or AAV8-Alb-sIl11ra1 virus to induce hepatocyte specific expression of mouse Il11ra1 or

soluble Il11ra1, respectively. As controls, both Il11ra1-/- mice and their wildtype littermates (Il11ra1+/+) were intravenously injected with 4X1011 gc AAV8-Alb-Null virus. 3 weeks after virus injection, mice were fed with HFMCD, WDF, or NC. Durations of diet are outlined in the main text and/or figure legends.
In vivo administration of soluble gp130
6-8-week old male C57BL/6N mice (InVivos) were injected with 4X1011 gc AAV8-Alb-sgp130 virus to induce hepatocyte specific expression of soluble gp130; control mice were injected with 4X1011 gc AAV8-Alb-Null virus. 3 weeks following virus administration, mice were fed with HFMCD, WDF, or NC for durations that are outlined in the main text and/or figure legends.
Il11ra-floxed mice (CKO)
Il11ra-floxed mice, in which exons 4 to 7 of the Il11ra1 gene were flanked by loxP sites, were created using CRISPR/Cas9 system as previously described26. To induce the specific deletion of Il11ra1 in hepatocytes, 6-8-week old male homozygous Il11ra1-floxed mice were intravenously injected with AAV8-Alb-Cre virus (4x1011gc); a similar amount of AAV8-Alb-Null virus were injected into homozygous Il11ra1-floxed mice as controls. The AAV8-injected mice were allowed to recover for three weeks prior to HFMCD, WDF, or NC feeding. Knockdown efficiency was determined by Western blotting of hepatic IL11RA.

Wild animals	The study did not involve wild animals.
Field-collected samples	The study did not involve field collected animals.
Ethics oversight	Animal experiments were performed under the guidelines of SingHealth Institutional Animal Care and Use Committee (IACUC).

Note that full information on the approval of the study protocol must also be provided in the manuscript.

Flow Cytometry

Plots

Confirm that:

- [x] The axis labels state the marker and fluorochrome used (e.g. CD4-FITC).
- [x] The axis scales are clearly visible. Include numbers along axes only for bottom left plot of group (a 'group' is an analysis of identical markers).
- [x] All plots are contour plots with outliers or pseudocolor plots.
- [ ] A numerical value for number of cells or percentage (with statistics) is provided.

Methodology

Sample preparation	For surface IL11RA, IL6R, and gp130 analysis, primary human hepatocytes and THP-1 cells were stained with IL11RA, IL6R, or gp130 antibody and the corresponding Alexa Fluor 488 secondary antibody. Cell death analysis was performed by staining primary human hepatocytes with Dead Cell Apoptosis Kit with Annexin V FITC and PI (V13242, Thermo Fisher).
Instrument	Fortessa, BD Biosciences
Software	FlowJo version X software (TreeStar)
Cell population abundance	3x10^5 cells/treatment group were used for each assay.
Gating strategy	The preliminary FSC/SSC gates of the starting cell population was 10,000 events. Debris (SSC-A vs FSC-A) and doublets (FSC-H vs FSC-A) were excluded. Boundaries between "positive" and "negative" staining were set at 10^3 for PI staining.

- [x] Tick this box to confirm that a figure exemplifying the gating strategy is provided in the Supplementary Information.
