# Supplementary material for: Hepatocyte-specific IL11 cis-signaling drives lipotoxicity and underlies the transition from NAFLD to NASH
Source: Nat Commun. 2021 Jan 4;12:66. doi: 10.1038/s41467-020-20303-z (PMC7782504; doi:10.1038/s41467-020-20303-z)

Uncropped western blot images

Figure 1f

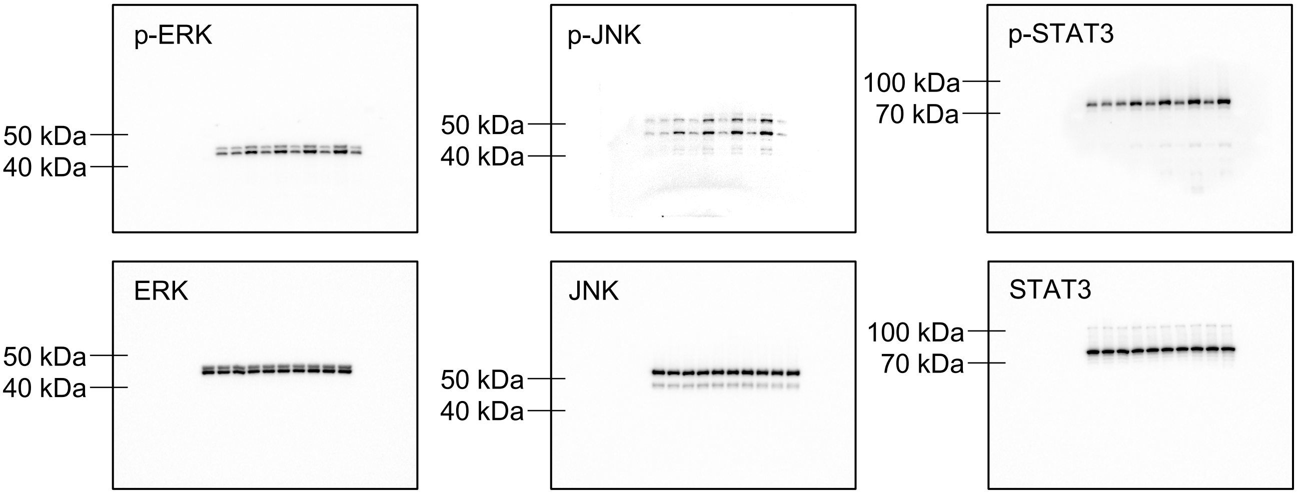

Figure 1i

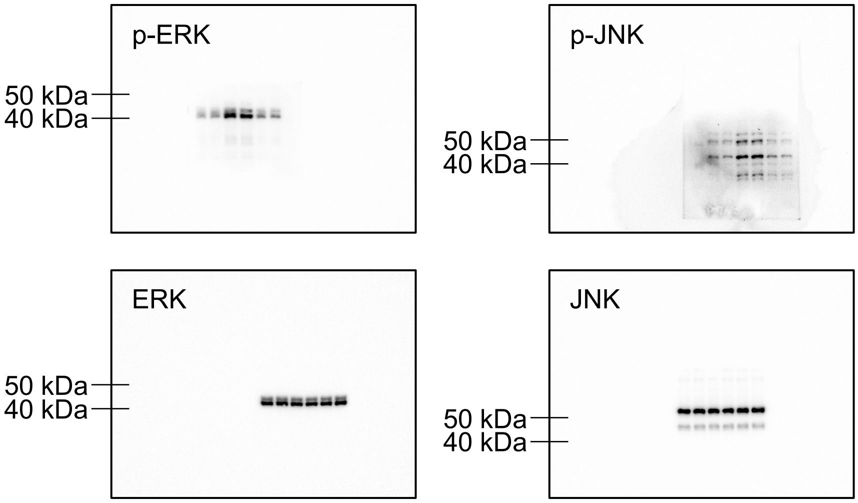

Figure 1j

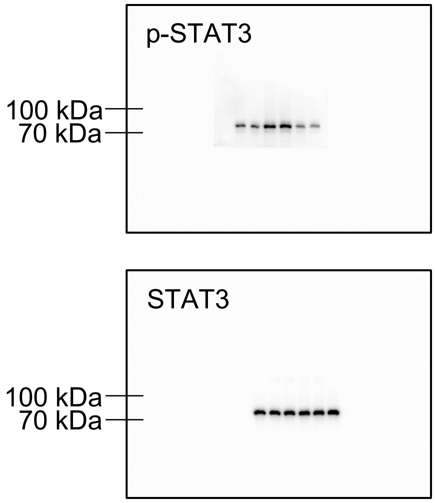

Figure 1l

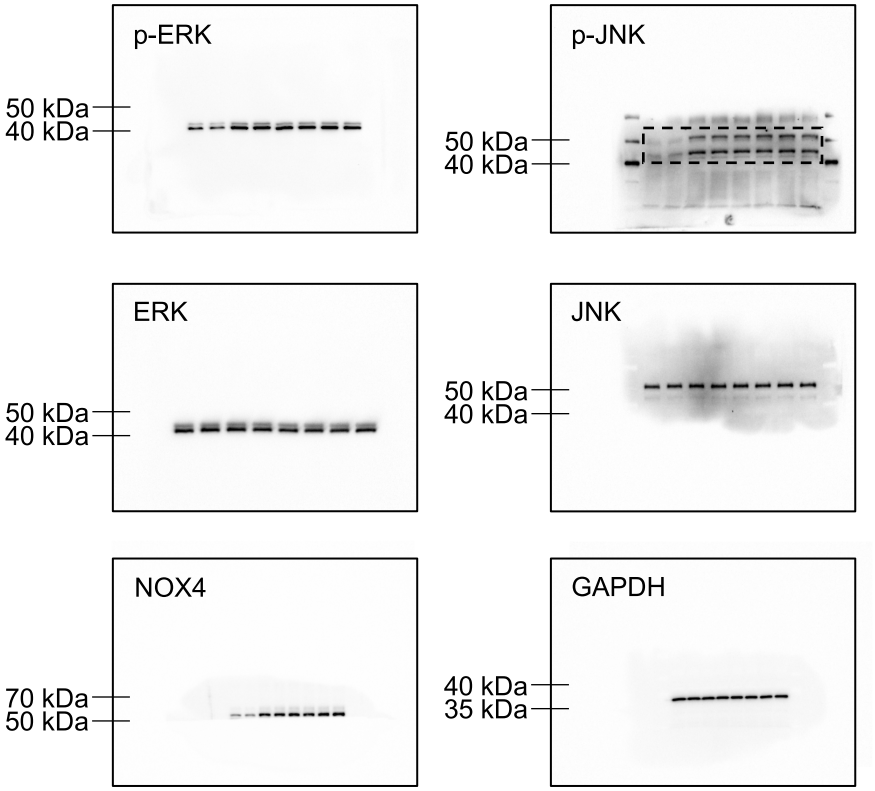

Figure 2j

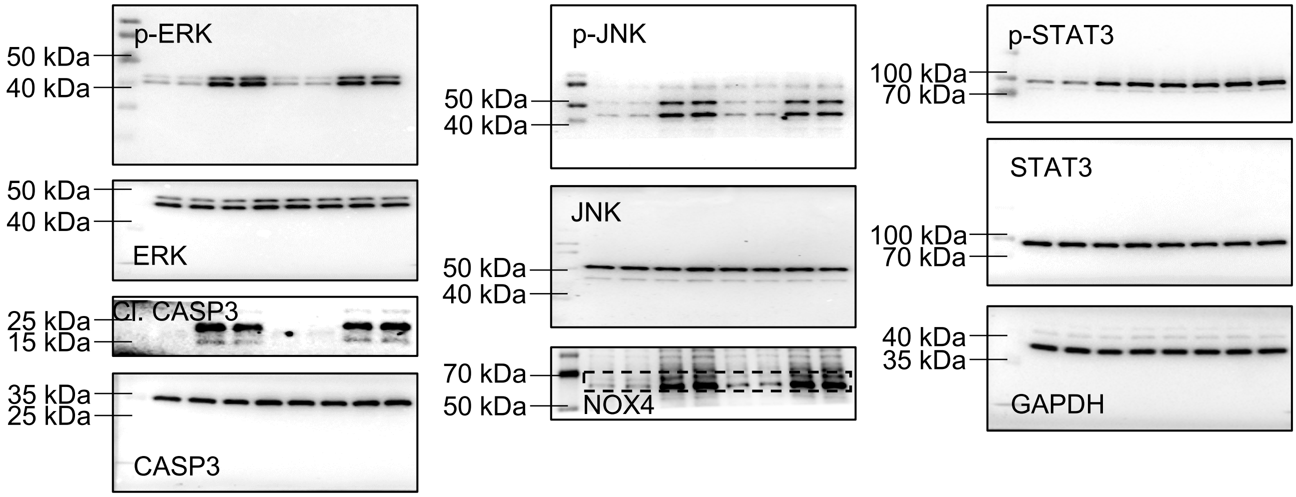

Figure 3b

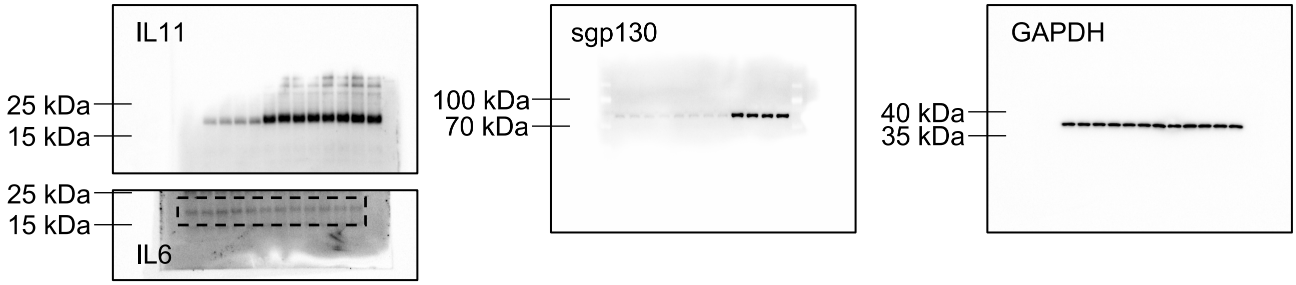

Figure 3p

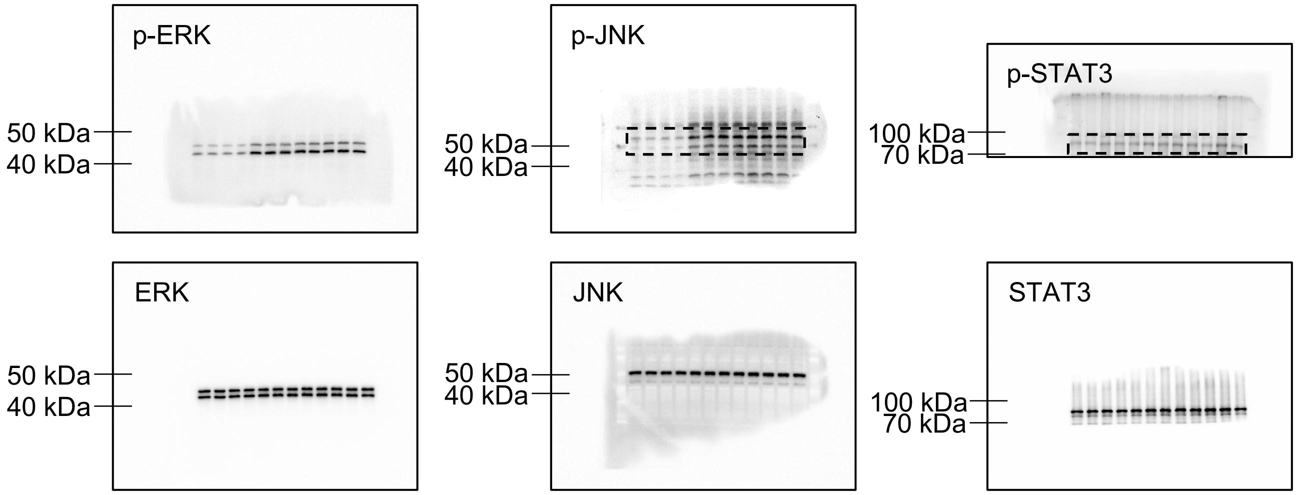

Figure 4b

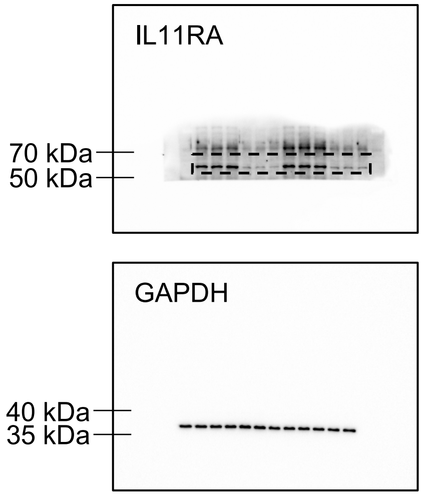

Figure 4k

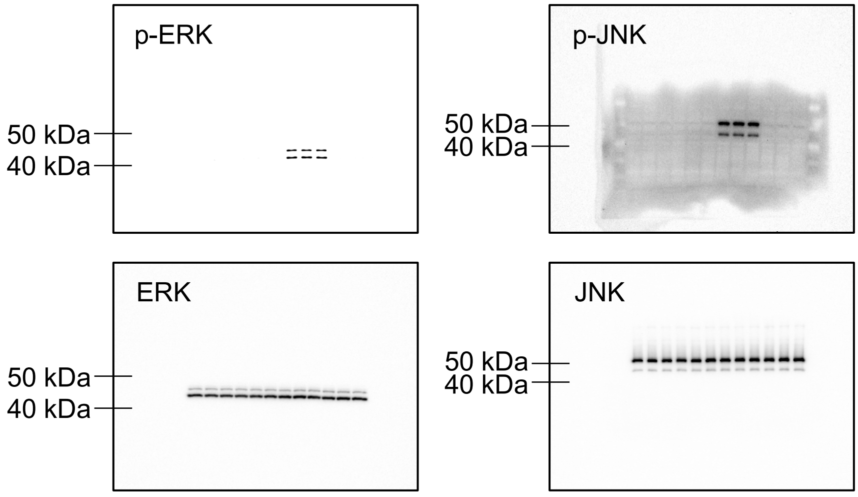

Figure 5b

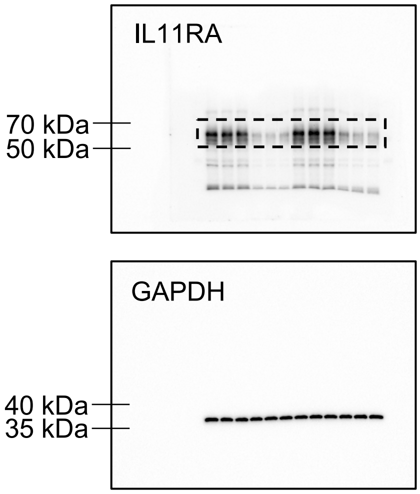

Figure 5m

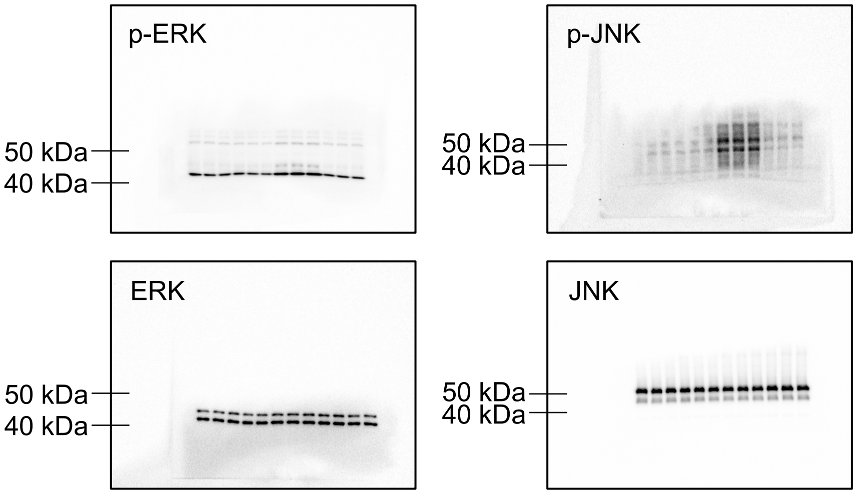

Figure 6b

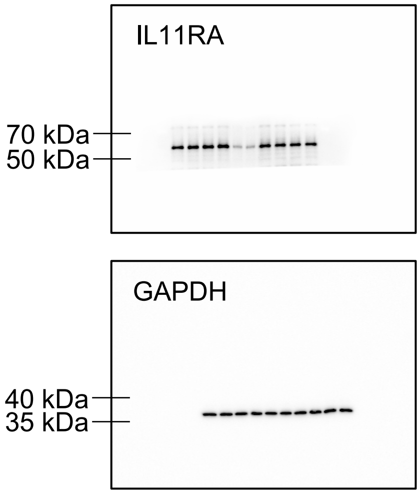

Figure 6k

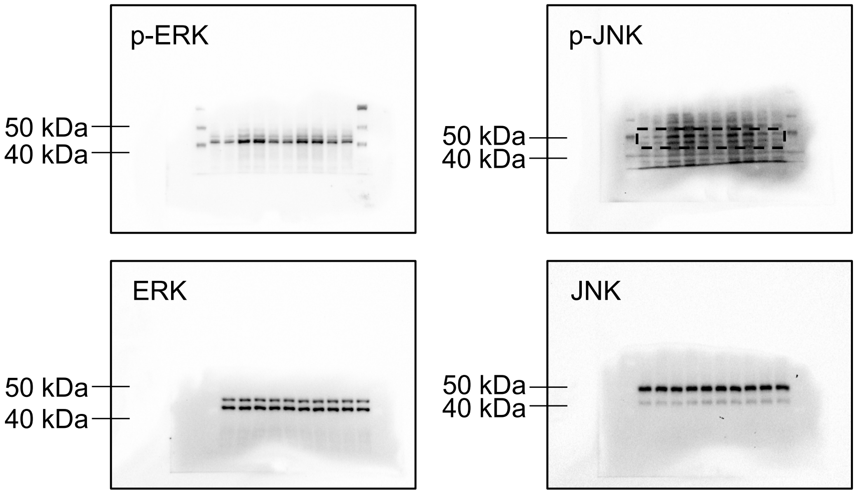

Supplementary Fig. 4a

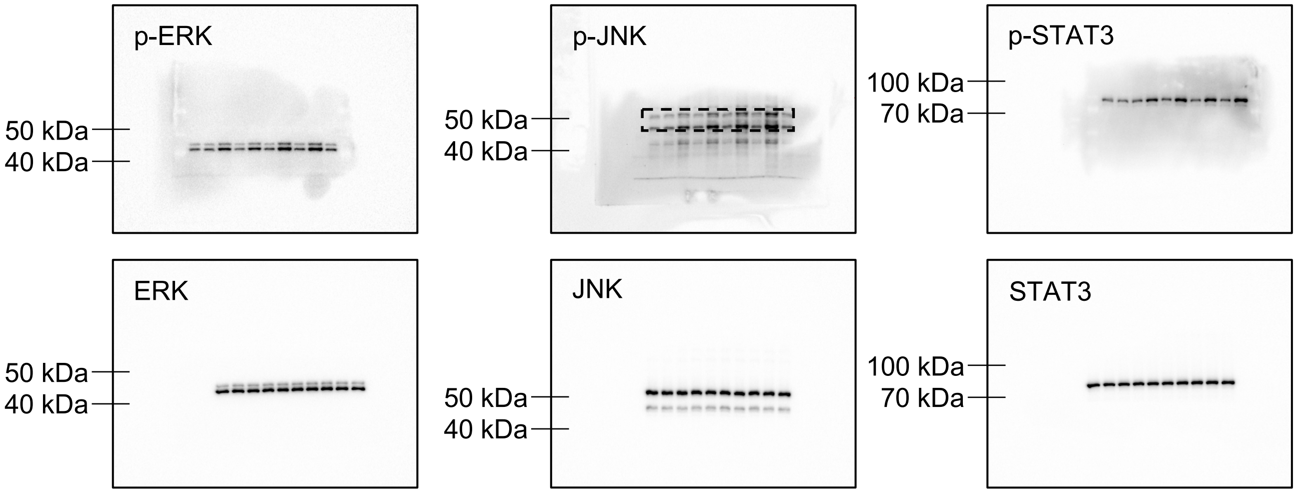

Supplementary Fig. 4e

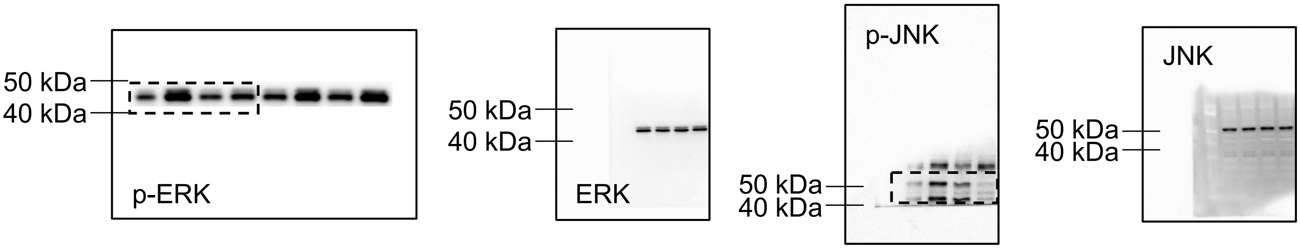

Supplementary Fig. 4g

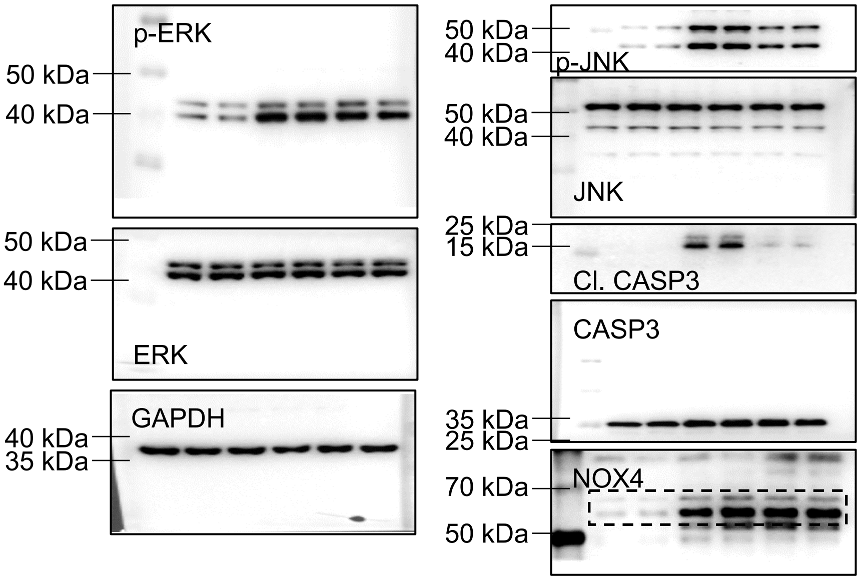

Supplementary Fig. 5c

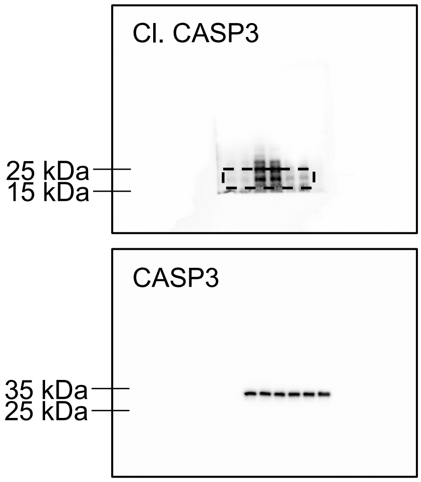

Supplementary Fig. 7b

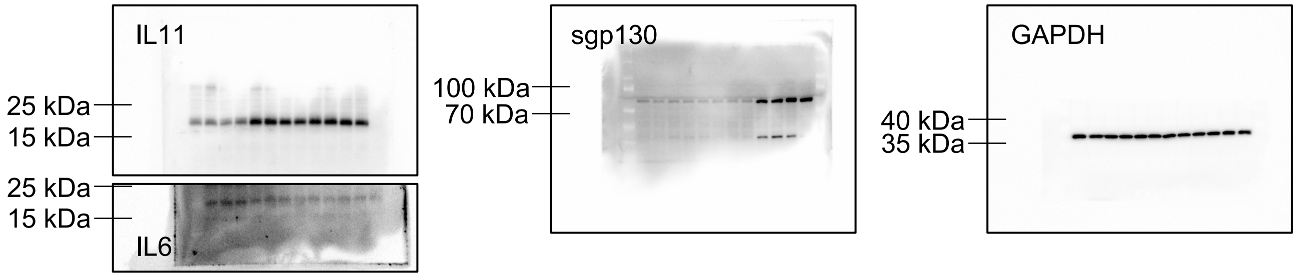

Supplementary Fig. 7n

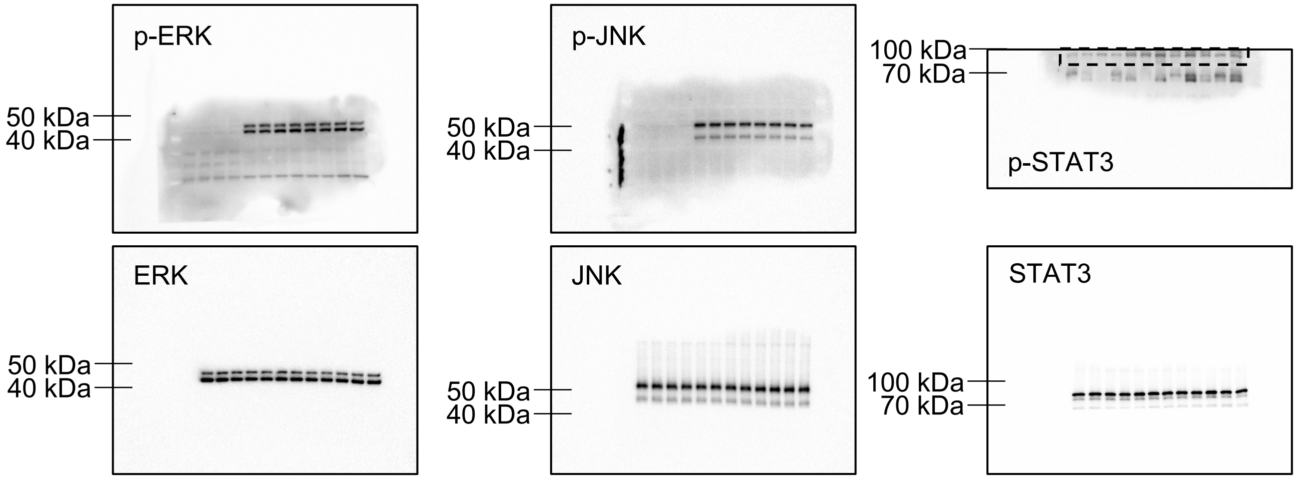

Supplementary Fig. 11b

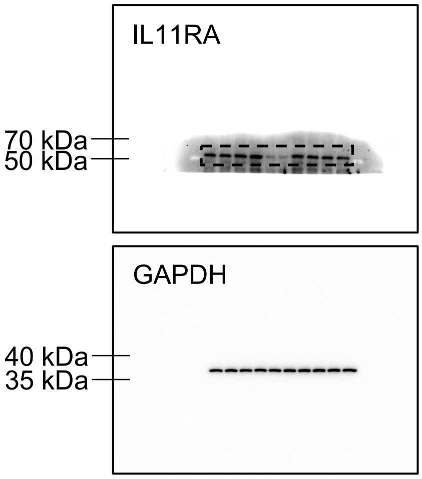

Supplementary Fig. 11l

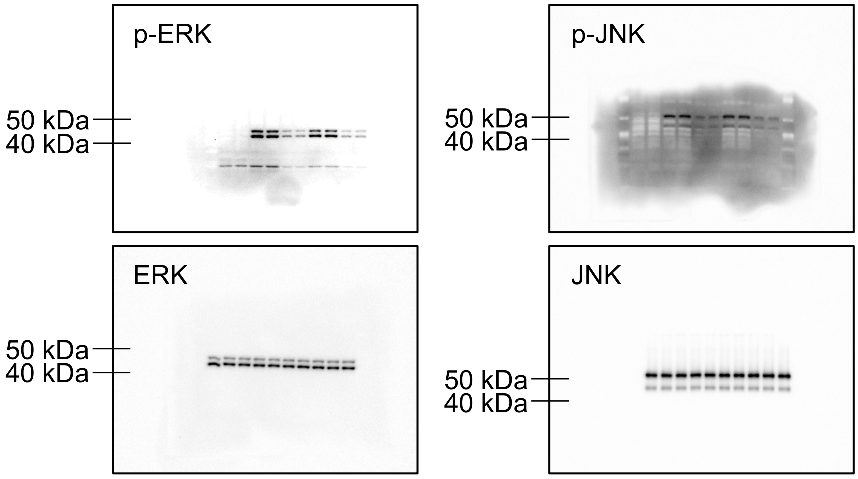

Supplement: Supplementary file 4 — Source Data [file 41467_2020_20303_MOESM4_ESM.zip › Source Data file 2 - Uncropped images.pdf]
